# Language impairment associated with prognosis in progressive supranuclear palsy

**DOI:** 10.1002/alz.090930

**Published:** 2025-01-03

**Authors:** Indira Ruth Garcia Cordero, Mohsen Hadian, Ece Bayram, Federico Rodriguez‐Porcel, Christopher Stephen, Alex Pantelyat, Jay Iyer, Tao Xie, Adam L Boxer, Douglas Gunzler, Marian Dale, Nikolaus McFarland, Kyurim Kang, Matthew Swan, Anne‐Marie Wills, Anthony E Lang, Maria Carmela Tartaglia

**Affiliations:** ^1^ Tanz Centre for Research in Neurodegenerative Diseases, University of Toronto, Toronto, ON Canada; ^2^ University of California San Diego, La Jolla, CA USA; ^3^ Medical University of South Carolina, Charleston, SC USA; ^4^ Department of Neurology, Massachusetts General Hospital and Harvard Medical School, Boston, MA USA; ^5^ Johns Hopkins University School of Medicine, Baltimore, MD USA; ^6^ Departments of Molecular and Cellular Biology and Statistics, Harvard University, MA, MD USA; ^7^ 4Department of Neurology, University of Chicago Medicine, Chicago, IL USA; ^8^ Memory and Aging Center, UCSF Weill Institute for Neurosciences, University of California, San Francisco, San Francisco, CA USA; ^9^ Population Health and Equity Research Institute, Center for Health Care Research and Policy, MetroHealth Medical Center, School of Medicine, Case Western Reserve University, Cleveland, Cleveland, OH USA; ^10^ Department of Neurology, Oregon Health & Science University, Portland, OR USA; ^11^ Department of Neurology, Norman Fixel Institute for Neurological Diseases, College of Medicine, University of Florida, Gainesville, FL USA; ^12^ Music and Health Science Research Collaboratory (MaHRC), Faculty of Music, University of Toronto, Toronto, ON Canada; ^13^ Department of Neurology, Mount Sinai Beth Israel, and Icahn School of Medicine, New York, NY USA; ^14^ Massachusetts General Hospital, Boston, MA USA; ^15^ Rossy PSP Program, University Health Network and the University of Toronto, Toronto, ON Canada; ^16^ The Edmond J. Safra Program in Parkinson’s Disease and Morton and Gloria Shulman Movement Disorders Clinic, Toronto, ON Canada; ^17^ Toronto Western Hospital, Tanz Centre for Research in Neurodegenerative Disease, Toronto, ON Canada

## Abstract

**Background:**

Language impairment is common in progressive supranuclear palsy (PSP) and is often overlooked due to the severity of the motor symptoms. We investigated whether language can be used to predict PSP prognosis.

**Methods:**

One hundred‐forty‐six patients with a diagnosis of possible or probable PSP from the Tilavonemab (ABBV‐8E12) clinical trial were evaluated at baseline and week 32 using the Repeatable Battery for the Assessment of Neuropsychological Status (RBANS), the PSP rating scale (PSPRS) and the Schwab and England Activities of Daily Living Scale (SEADL). Percentage of change was calculated for each measure. Using correlations, we evaluated relationships between all RBANS‐subscores (language, attention, memory and visuoconstructional), the PSPRS and SEADL scores at baseline; p‐values were FDR corrected. Linear regression analyses were performed between the RBANS‐language, RBANS‐delayed‐memory and RBANS‐executive at baseline and the percentage of change over time. Grey matter volumes were extracted from three regions of interest based on language (bilateral temporal poles and inferior frontal gyri), executive (bilateral dorsolateral and superior frontal gyri and frontal pole) and memory (bilateral hippocampus, inferior, middle and superior temporal gyri) areas.

**Results:**

Mean age of PSP patients: 68.8 years (49‐86 years), 57 (39%) females. The RBANS language, executive and delayed memory scores at baseline were positively correlated with each other and with visuoconstructional and immediate memory score (all p<0.05). Only the RBANS‐language score at baseline predicted percentage of change in PSPRS (B = ‐0.63, p = 0.003). The percentage of change in the RBANS‐language score was predicted by the RBANS‐language at baseline (B = ‐0.59, p<0.001). Lower age at baseline was associated with a worsening in language score over time (B = 0.51, p = 0.009). Language grey matter volume was associated with the change in RBANS‐language score (B = 0.01, p = 0.02).

**Conclusions:**

language impairment at baseline, in contrast to memory and executive functions, was predictive of functional decline as measured by PSPRS. Atrophy in language areas at baseline predicted language decline. Language impairment may be an independent prognostic factor in PSP.

*Based on research using data from AbbVie that has been made available through Vivli, Inc. Vivli has not contributed to or approved, and is not in any way responsible for, the contents of this publication.

